# A 14-Year-Old Male Patient with Kawasaki Disease Presented with Stroke after COVID-19

**DOI:** 10.1155/2021/5576440

**Published:** 2021-06-12

**Authors:** Nexhmedin Shala, Fisnik Jashari, Dren Boshnjaku, Argjend Shala, Pranvera Ibrahimi, Vera Kukaj, Shemsedin Dreshaj

**Affiliations:** ^1^Clinic of Neurology, University Clinical Center of Kosovo, Pristina 10000, Kosovo; ^2^Department of Neurology, Faculty of Medicine, University of Pristina “Hasan Prishtina”, Pristina 10000, Kosovo; ^3^Clinic of Cardiology, University Clinical Center of Kosovo, Pristina 10000, Kosovo; ^4^UBT College, Institute of Medical Sciences and Innovation, Pristina 10000, Kosovo; ^5^Clinic of Infectious Disease, University Clinical Center of Kosovo, Pristina 10000, Kosovo; ^6^Department of Infectious Disease, Faculty of Medicine, University of Pristina “Hasan Prishtina”, Pristina 10000, Kosovo

## Abstract

According to several studies, children represent only about 2% of the patients affected by the current SARS-CoV-2, and most often, they are asymptomatic. However, there is a concern about a vascular inflammatory disease which is similar to Kawasaki disease observed in children and adolescents weeks after infection. We report a case of Kawasaki disease presented with ischemic stroke in a 14-year-old male patient following SARS-Cov-2 infection.

## 1. Introduction

The COVID-19 pandemic has resulted in over hundred million confirmed cases and millions of deaths. Studies have shown that pediatric patients represent a small percentage of confirmed cases and they display only mild symptoms, making the diagnosis challenging [1, 2]. However, several reports from European countries and North America have described a secondary Multisystem Inflammatory Syndrome in Children (MIS-C) and also patients in their late teens and early twenties who have presented with features of Kawasaki disease (KD) after prior COVID-19 [3]. This alarming complication of COVID-19 in children usually occurs between two and four weeks after infection. Majority of children and adolescents affected by this complication were previously healthy, about 80% of them required intensive care, 2% have died, and many of them developed cardiovascular and clotting complications [4, 5]. Here, we report a clinical case showing an association between SARS-CoV-2 infection and typical KD presented with ischemic stroke. KD affects medium- and small-sized arteries, mainly coronary arteries, and cases complicated with stroke are rarely identified and reported in the literature [6]. To the best of our knowledge, this is the first report of a patient with KD presented with ischemic stroke after COVID-19.

## 2. Case Description

**Figure 1:**
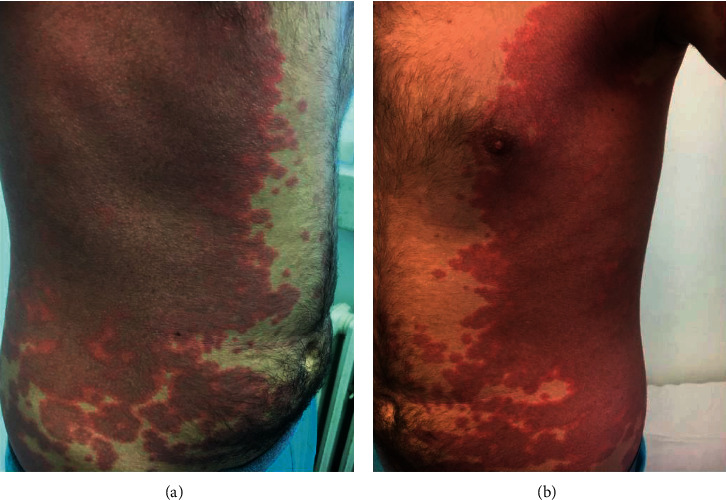
Very prominent skin rashes affecting the lateral parts of the body in both sides.

**Figure 2:**
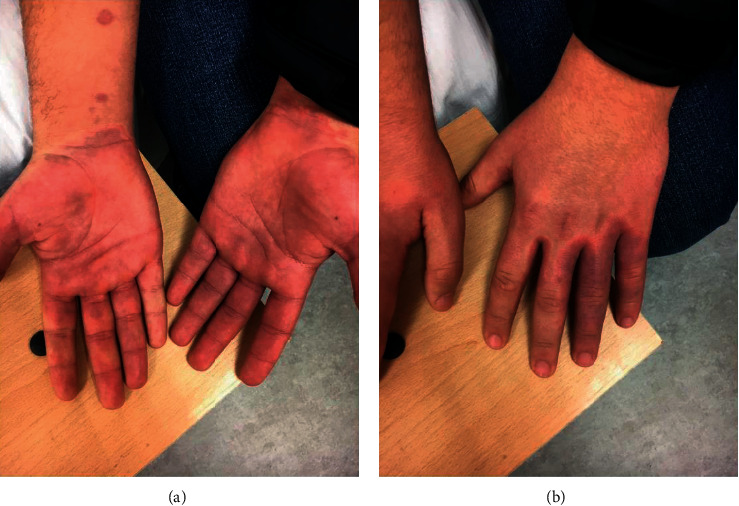
Skin rashes in the distal parts of the upper extremities and palmar edema.

**Figure 3:**
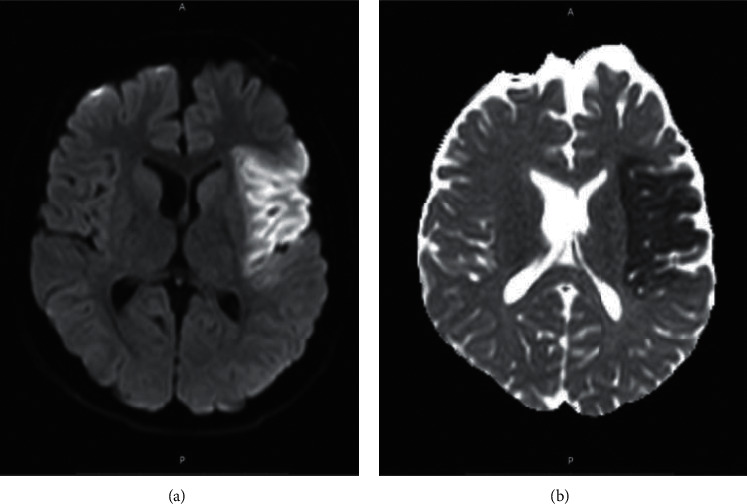
Axial MRI images (a) diffusion weighted and (b) apparent diffusion coefficient (ADC), showing acute brain ischemia in the territory of the medial cerebral artery.

A 14-year-old male with no chronic medical conditions presented in the Emergency Department with a five-day history of fever, fatigue, bilateral conjunctivitis, cervical lymphadenopathy, hand swelling, and a skin rash on his lateral parts of the body, groin, and both hands (Figures 1 and 2). Because the skin rashes were very prominent, the patient was at first admitted in the clinic of dermatology, and for seven days, he was treated with empirical antibiotics and corticosteroid therapy. He did not develop any significant lower respiratory symptoms. On the third day of being in hospital, his fever resolved and skin rashes began to resolute. On the seventh day of being in hospital, he developed right-side hemiplegia and aphasia. Brain MRI (Magnetic Resonance Imaging) and MRA (Magnetic Resonance Angiography) were conducted, showing brain ischemia on the terminal branches of the left medial cerebral artery (Figure 3). As a result, the patient was admitted in our Clinic of Neurology for further evaluation and treatment.

His laboratory testing showed increased white blood cell count (WBC) 13 100/mm^3^ with increased granulocytes, normal hemoglobin and thrombocyte levels, elevated inflammatory markers (ESR 65 mm/min, CRP 85.1 mg/L), increased d-dimer (4.8 mcg FEU/ml), low albumin level (31.8 g/L), and normal transaminases, bilirubin, blood urea nitrogen, and creatinine level. The patient had mild pyuria (5–10 WBC/hpf), but there was no pathogen isolated in the urine culture. The lumbar puncture was performed showing slight increase of proteins (0.92 g/L) and no blood elements. A plain view chest radiograph showed no significant abnormalities. Electrocardiography showed normal sinus rhythm. Transthoracic echocardiography showed normal ejection fraction without coronary artery dilatation. Brain and neck Computed Tomography Angiography (CTA) was normal. However, on transcranial duplex ultrasound, a slight increase of the pulsatility index (PI = 1.18) was observed, suggesting distal resistance due to occlusion of the terminal arterial branches of the left medial cerebral artery (MCA). An autoimmunity panel screening was performed, and the results were negative.

Three weeks before symptom onset, the patients' mother had been confirmed with COVID-19 and displayed mild upper respiratory infection, but our patient was not tested at that time as he had not exhibited fever or any other symptoms of illness. After admission in our hospital, the nasopharyngeal SARS-CoV-2 PCR was negative but serological testing for COVID-19 showed increased IgG levels. Serological (IgM) screening for cytomegalovirus, toxoplasmosis, rubella, HIV, and herpes virus was negative.

**Figure 4:**
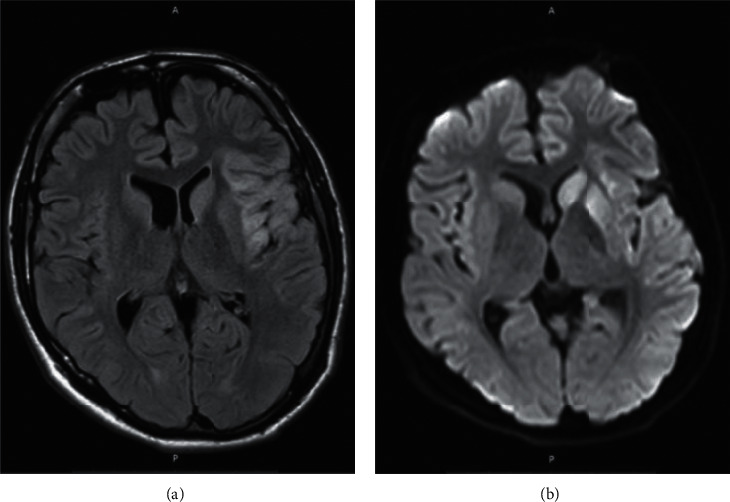
Brian MRI images of the patient, two weeks after neurological symptom onset. Axial (a) fluid-attenuated inversion recovery (FLAIR) MRI scans and (b) diffusion-weighted images (DWI) showing a hyperintense signal and edema of the caudate nucleus head, putamen, and parts of the external capsule and insula on the left side.

KD was diagnosed when our patient met four out of five clinical criteria (generalized rash, peripheral extremity swelling, bilateral nonexudative conjunctivitis, and cervical lymphadenopathy) associated with more than five days of fever. Intravenous immunoglobulin (IVIG) (2 g/Kg) was administrated together with a low dose of aspirin therapy and methylprednisolone 40 mg twice daily. Within 48 hours, the patient improved significantly with near complete resolution of neurological symptoms, and he was able to speak and move, showing only discrete right-side hemiparesis (4/5 muscle strength). Prior to hospital discharge, two weeks later, complete blood count, coagulation profile, d-dimer, albumin level, and CRP were normalized. Also, ischemic changes on brain imaging were decreased (Figure 4). On the follow-up visit, two weeks after hospital discharge, the patient continued to do well without recurring symptoms.

## 3. Discussion

On 27 April 2020, the United Kingdom National Health Service issued an alert highlighting a multisystem inflammatory syndrome increasingly observed across the United Kingdom, citing a possible association with the SARS-CoV-2. Authors from London, England, [3] and authors from Philadelphia, USA, [7] reported clinical and laboratory features of several patients aged 2–15 years who had been hospitalized with hyperinflammatory shock, all of whom tested positive for SARS-CoV-2 antibodies. Clinical characteristics of these cases shared features with KD and KD shock syndrome, including fever, shock, and variably rash, conjunctivitis, extremity edema, and gastrointestinal symptoms. Centers for Disease Control and Prevention issued a public health advisory and case definition for this hyperinflammatory syndrome, termed MIS-C [8]. They recommended that early diagnosis and treatment of patients meeting full or partial criteria for KD is critical to prevent complications. Patients meeting criteria for KD should be treated with intravenous immunoglobulin (IVIG) and aspirin [9].

Here, we have reported a 14-year-old patient with classical signs of KD presented with ischemic stroke. The diagnosis was challenging because the prior COVID-19 was silent and the skin rashes were very prominent on admission. Therefore, the patient was admitted and empirically treated in the clinic of dermatology at first days, and only after complicated with neurological symptoms, he was transferred to the Neurology Clinic. Prior infection with SARS-CoV-2 was confirmed based on serological testing and prior contact with the infected family member. Brain ischemia was confirmed with MRI. Even though there was not any large artery or venous occlusion apparent on CTA, increased pulsatility index was found on the left MCA suggesting distal small diameter arterial segments occlusion. After treatment with IVIG, corticosteroids, and low-dose aspirin, the neurological deficit was resolved.

The exact pathomechanism of the MIS-C is not clear. Given the lag between SARS-CoV-2 infection and MIS-C and the finding that many patients are positive for antibodies and negative for the viral antigen, it has been suggested that abnormal immune response might be the key factor. A potential role of the antibodies in the pathogenesis of KD has been accepted, and the prevalence of such antibodies to target different tissues in the body could explain the multisystem presentation in MIS-C [10].

## 4. Conclusions

There is a myriad of COVID-19 symptoms and sequelae making the diagnosis challenging. Early diagnosis and prompt treatment of the secondary inflammatory syndromes, including Kawasaki disease, is very important for preventing end-organ damage and long-term complications.

## Data Availability

The data that support the findings of this study are available from the corresponding author upon reasonable request.
